# The Complex Interplay between Metabolic Reprogramming and Epigenetic Alterations in Renal Cell Carcinoma

**DOI:** 10.3390/genes10040264

**Published:** 2019-04-02

**Authors:** Ana Lameirinhas, Vera Miranda-Gonçalves, Rui Henrique, Carmen Jerónimo

**Affiliations:** 1 Cancer Biology & Epigenetics Group—Research Center, Portuguese Oncology Institute of Porto (CI-IPOP), 4200-072 Porto, Portugal; analameirinhas@gmail.com (A.L.); vera.miranda.goncalves@ipoporto.min-saude.pt (V.M.-G.); rmhenrique@icbas.up.pt (R.H.); 2 Master in Oncology, Institute of Biomedical Sciences Abel Salazar, University of Porto (ICBAS-UP), 4050-313 Porto, Portugal; 3 Department of Pathology, Portuguese Oncology Institute of Porto, 4200-072 Porto, Portugal; 4 Department of Pathology and Molecular Immunology, Institute of Biomedical Sciences Abel Salazar– University of Porto (ICBAS-UP), 4050-313 Porto, Portugal

**Keywords:** renal cell carcinoma, metabolic reprograming, Warburg effect, epigenetic alterations

## Abstract

Renal cell carcinoma (RCC) is the most common malignancy affecting the kidney. Current therapies are mostly curative for localized disease, but do not completely preclude recurrence and metastization. Thus, it is imperative to develop new therapeutic strategies based on RCC biological properties. Presently, metabolic reprograming and epigenetic alterations are recognized cancer hallmarks and their interactions are still in its infancy concerning RCC. In this review, we explore RCC biology, highlighting genetic and epigenetic alterations that contribute to metabolic deregulation of tumor cells, including high glycolytic phenotype (Warburg effect). Moreover, we critically discuss available data concerning epigenetic enzymes’ regulation by aberrant metabolite accumulation and their consequences in RCC emergence and progression. Finally, we emphasize the clinical relevance of uncovering novel therapeutic targets based on epigenetic reprograming by metabolic features to improve treatment and survival of RCC patients.

## 1. Introduction

Renal cancer (RC) is the 16th most incident cancer worldwide according to GLOBOCAN 2018 estimates, accounting for approximately 2% of all cancer-related deaths [1].

Cigarette smoking, obesity and hypertension are the main recognized risk factors for RC [2], although other medical conditions, including renal lithiasis, urinary tract infection and chronic kidney disease, have also been associated with increased risk [3]. Sex also affects RC incidence and mortality, as this malignancy is 50% less frequent in women compared to men [4], which might be due to different smoking habits, occupational risk factors and sex steroid hormones [5]. Moreover, genetics factors might also lead to this two-fold increased risk in men [6]. Approximately 2–3% of all RC are associated with familial history, and several distinct genetic alterations are described [7]. Renal cell carcinoma (RCC) is the most common RC type [7], with clear cell RCC (ccRCC) subtype encompassing approximately 75% of all RCC, followed by papillary RCC (pRCC) and chromophobe RCC (chRCC), which constitute approximately 10% and 5% of all cases, respectively [8]. This review focuses on the genetic and epigenetic alterations disclosed by RCC that have been implicated in aberrant tumor metabolism. Furthermore, the interactions between intracellular metabolites and epigenetic mechanisms are also addressed.

## 2. Clear Cell Renal Cell Carcinoma

Clear cell RCC might be sporadic or hereditary [9], the latter comprising a small percentage of the total of ccRCC cases, whereas sporadic ccRCC represents more than 80% of the diagnosed cases [7]. Most familial ccRCC are associated with von Hippel Lindau disease, caused by von Hippel Lindau tumor suppressor gene (*VHL*) genetic alterations, at chromosome 3p (3p25–26) [10]. Furthermore, BRCA1 associated protein 1 (*BAP1*) and succinate dehydrogenase (*SDHB*, *SDHC* and *SDHD*) gene mutations have also been associated with ccRCC hereditary forms [10].

Remarkably, 90% of sporadic ccRCC cases demonstrate loss of chromosome 3p, which is related with *VHL* gene alteration [11]. Indeed, biallelic *VHL* inactivation caused by genetic mutations and/or by *VHL* promoter hypermethylation is considered a driving event in ccRCC carcinogenesis [12]. The VHL protein is part of ubiquitin ligase complex responsible of directing hypoxia-inducible factors (HIF) 1α and 2α for degradation [13,14]. Under normoxic conditions, HIFs suffer hydroxylation on conserved proline residues by prolyl hydroxylase (PHD) proteins and the HIF-α hydroxylated proteins enable VHL complex binding, which targets these proteins for ubiquitination and proteasomal degradation [15]. Under hypoxia, HIF-α is not hydroxylated by PHD as these proteins are inactive [16]. Consequently, HIF-α accumulates in the nucleus, resulting in activation of its target genes, which are implicated in the regulation of glycolysis, cell proliferation and angiogenesis [17]. In ccRCC, *VHL* loss leads to pseudo-hypoxic condition and consequent abnormal accumulation of HIF proteins, although oxygen levels remain normal for these cells [18,19].

Along with *VHL* loss, mutations in other genes located at chromosome 3p have also been reported in ccRCC [11]. These include mutations in chromatin remodeling genes, e.g., polybromo 1 (*PBRM1*), SET domain containing 2 (*SETD2*) and *BAP1*, as well as activating mutations in mammalian target of rapamycin (mTOR) pathway, which are prevalent in ccRCC and associated with worse clinical outcome [11].

## 3. Papillary Renal Cell Carcinoma

Papillary RCC comprises a heterogeneous group of cancers with distinct histological and cytogenetical characteristics, and rather divergent disease progression and outcome [7]. Similar to ccRCC, pRCC usually occurs in sporadic forms, but hereditary syndromes are also implicated in pRCC development [20]. These tumors are histopathologically categorized in two major subtypes, pRCC Types I and II [21]. Type I is less frequent and may have better prognosis, compared with Type II [22]. Besides, it is typically associated with MET proto-oncogene, receptor tyrosine kinase (*MET*) amplification and overexpression [23,24]. On the other hand, hereditary papillary renal cancer (HPRC), the familiar form of pRCC Type I, is characterized by activating mutations in the tyrosine kinase domain of *MET*, which is located at chromosome 7p [25,26]. *MET* encodes for a cell surface receptor to which hepatocyte growth factor (HGF) binds, activating several pathways responsible for cell proliferation, survival and morphogenesis [27]. Besides *MET* mutations, pRCC Type I is also associated with gains in chromosomes 3q, 7, 12, 17, and 20 [28].

Contrarily to Type I, *MET* mutations are rare in Type II pRCC, but gene amplification may be observed, although in a small number of cases [21]. Sporadic Type II tumors are frequently associated with cyclin dependent kinase inhibitor (*CDKN*) *2A* inactivation, either by mutation or promoter hypermethylation. Additionally, mutations of chromatin remodeling genes (*SETD2*, *BAP1* and *PBRM1* genes) described for ccRCC have also been found [21]. The familiar form arises from hereditary leiomyoma renal cell carcinoma (HLRCC) syndrome and it due to fumarate hydratase (*FH*) gene germline mutations [29]. FH-deficient tumors display fumarate accumulation that inhibit PHD activity, resulting in a pseudo-hypoxic environment due to HIF-α accumulation [30]. Thus, loss of *FH* expression associated with hypoxia-related genes accumulation with consequent tumor cell progression and glycolytic metabolism [21,31].

## 4. Tumor Metabolism

### 4.1. Warburg Effect

Over the last decades, remarkable progress in the knowledge of carcinogenesis and tumor biology has been accomplished, resulting from the recognition of new cancer hallmarks, such as reprogramming of energy metabolism [32]. Indeed, metabolic switch is a well substantiated mechanism essential for tumor initiation and progression, as it supports cell growth and division [32].

Under aerobic conditions, normal quiescent cells of differentiated tissues convert glucose into pyruvate via glycolysis, which is transported from the cytosol to the mitochondria, and subsequently converted to acetyl-coenzyme A (acetyl-CoA). Thereafter, acetyl-CoA enters the tricarboxylic acid cycle (TCA) and oxidative phosphorylation (OXPHOS) occurs. In these conditions, cells show basal glycolysis rate and most of adenosine triphosphate (ATP) is provided by OXPHOS [33]. Conversely, in the absence of oxygen, glycolysis is favored and pyruvate is metabolized to lactate [32]. Proliferative cells undergo a metabolic switch, due to bioenergetic and biosynthesis needs. These cells display increased glucose consumption, as well as high glycolytic rates, lactate production, and lipids biosynthesis and of other macromolecules [34].

Tumor cells share the metabolic signature of proliferating cells to fuel cell growth and division [35]. In fact, the highly glycolytic cancer cell metabolism is sustained even in the presence of oxygen, so-called aerobic glycolysis or Warburg effect [35,36]. Thus, in tumor cells, most of the incoming glucose is converted into lactate (approximately 85%), and just a small percentage of pyruvate produced by glycolysis is metabolized in the mitochondria through OXPHOS (about 5%) [34]. Compared to mitochondrial OXPHOS, glycolysis is bioenergetically less efficient, generating less ATP than OXPHOS [37], but allows rapid ATP synthesis in the cytoplasm [38,39]. Furthermore, glycolysis is sustained by preservation of nicotinamide adenine dinucleotide (NAD)^+^/NADH ratio, since the consumed NAD^+^ is regenerated during pyruvate conversion to lactate [33,40]. In fact, metabolic reprogramming confers several advantages to tumor cells [41]. First, glycolysis supports biosynthesis of lipids, amino acids and also nucleotides by providing intermediates of the glycolytic pathway for anabolic reactions. Cancer energy metabolism promotes cell survival and proliferation under fluctuating oxygen availability. Additionally, Warburg effect protects cells from oxidative stress by reducing reactive oxygen species (ROS) production due to increased levels of antioxidant glutathione. Moreover, increased glycolysis results in augmented lactate production, contributing to microenvironment acidification, which has been associated with invasive phenotype, including increased migration, invasion, metastization, immunosuppression and therapy resistance [38,39,41].

Interestingly, an intra-tumoral symbiosis was described in these tumors. Considering the blood vessels distribution, the tumor cells are exposed to different O2 levels. Hypoxic cancer cells are more distant from the supplying blood vessel and are poorly oxygenated. These cells display high HIF-1α expression that induces glycolytic proteins expression, increasing glucose uptake and lactate production, as well as lactate efflux. On the other hand, well oxygenated tumor cells are able to perform lactate uptake and preferentially use it as the main energy source, by employing it as main substrate for mitochondrial OXPHOS [42,43]. Knowing that both tumor metabolic reprograming and intra-tumoral symbiosis have an important role in tumor growth and therapy resistance, new anti-cancer therapies targeting cancer bioenergetics have been developed.

### 4.2. Metabolic Players in Renal Cell Carcinoma 

**Figure 1:**
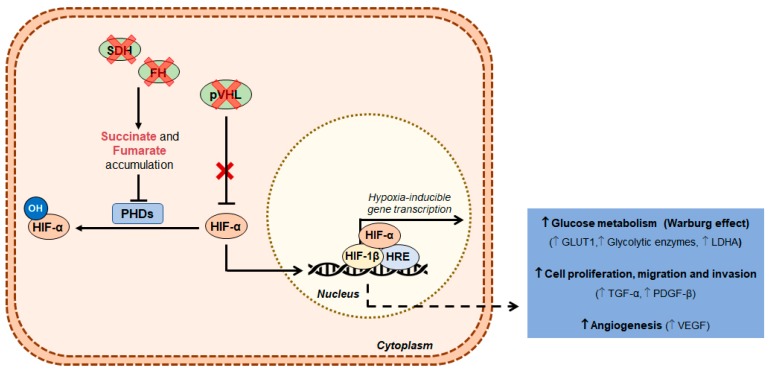
Genetic mutations, HIF-1α activation and metabolism of RCC. Succinate and fumarate accumulation, as well as VHL inactivation leads to increased glycolytic metabolism by HIF pathway activation. Abbreviations: FH, Fumarate hydratase; GLUT, Glucose transporter; HIF, Hypoxia-inducible factor; HRE, Hypoxia-response elements; LDH, Lactate dehydrogenase; PDGF, Platelet derived growth factor; PHD, Prolyl hydroxylases; pVHL, VHL protein; SDH, Succinate dehydrogenase; TGF, Transforming growth factor; VEGF, Vascular endothelial growth factor.

As described in the previous section, RCC is characterized by mutations in genes related with cell metabolism, namely *FH* and *SDH* genes. FH- and SDH-deficient RCC accumulate fumarate or succinate, respectively, which inhibits PHDs, leading to HIF stabilization and accumulation in the cytosol (Figure 1). In addition to *FH* and *SDH* mutations, loss of *VHL* function is also found in a large proportion of RCCs. Indeed, VHL is responsible for HIF degradation, so that in cells lacking VHL, HIF is stabilized, leading to HIF pathway activation (Figure 1) [44,45]. Furthermore, increased HIF translation may result from mTOR and MET activation [44]. Consequently, HIF-1α target genes, implicated in glucose metabolism, cell proliferation and angiogenesis, are upregulated, contributing to a glycolytic phenotype and cancer progression [46,47].

**Figure 2:**
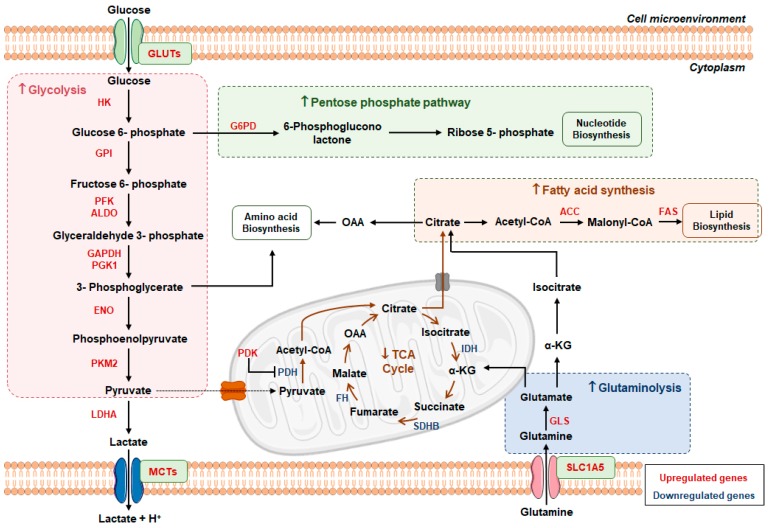
Metabolic reprogramming in RCC. RCCs are characterized by a high glycolytic phenotype with increased lactate production, where glucose is the major energy source. Pentose phosphate pathway, fatty acid synthesis and glutaminolysis are also increased to sustain nucleotides, amino acids and lipids biosynthesis. In contrast, TCA cycle activity is decreased in these tumors. In the scheme proteins that were found upregulated in RCC are labeled red, while downregulated proteins are labeled blue. Abbreviations: ACC, Acetyl-coenzyme A carboxylase; Acetyl-CoA, Acetyl-coenzyme A; ALDO, Fructose-bisphosphate aldolase; ENO, Enolase; FAS, Fatty acid synthase; G6PD, Glucose-6-phosphate dehydrogenase; GAPDH, Glyceraldehyde-3-phosphate dehydrogenase; GLS, Glutaminase; GPI, Glucose phosphatase isomerase; HK, Hexokinase; IDH, Isocitrate dehydrogenase; MCT, Monocarboxylate transporter; OAA, Oxaloacetate; PDH, Pyruvate dehydrogenase; PDK, Pyruvate dehydrogenase kinase; PFK, 6-phosphofructokinase; PGK, Phosphoglycerate kinase; PKM, Pyruvate kinase M; SLC1A5, Solute carrier family 1 member 5; TCA, Tricarboxylic acid cycle; α-KG, Alpha-ketoglutarate.

Interestingly, HIF-1 constitutive upregulation has been associated with increased transcription levels of genes encoding: (1) glucose transporters (*GLUT*) *1* and *3*; (2) glycolytic enzymes, e.g., hexokinases (*HK*) *2*; (3) glucose phosphatase isomerase (*GPI*); and (4) phosphoglycerate kinase (*PGK*) 1 [48]. Moreover, hypoxia-response elements might also upregulate lactate dehydrogenase (*LDH*) *A* and monocarboxylate transporter (*MCT*) *4*, suggesting that pyruvate produced via glycolysis is metabolized into lactate [46,49]. Additionally, high pyruvate dehydrogenase kinase (*PDK*) 1 expression levels have been reported in RCC, as this enzyme inhibits pyruvate dehydrogenase (PDH) activity responsible for the conversion of pyruvate to Acetyl-CoA, leading to TCA cycle deprivation [50]. Conversely, downregulation of respiratory complexes is associated with mitochondrial inactivity [48,50]. Thus, RCCs display increased glycolysis and lactate production along with reduced OXPHOS, which are characteristic of Warburg effect (Figure 2).

Besides glycolysis, other metabolic enzymes related to fatty acids metabolism, glutaminolysis and pentose phosphate pathway (PPP) were also found deregulated in RCC (Figure 2) [49,51,52]. Specifically, glucose-6-phosphate dehydrogenase (*G6PD*) overexpression increases PPP activity [53,54], which generates high nicotinamide adenine dinucleotide phosphate (NADPH) levels. This is not only critical to oxidative stress, apoptosis and radiation protection, but it also provides ribose molecules required for nucleotide biosynthesis [53]. Additionally, increased levels of acetyl-CoA carboxylase (ACC) and fatty acid synthase (FAS) (enzymes of the fatty acids synthesis pathway) were associated with RCC aggressiveness and poor prognosis [11,55]. Remarkably, glutamine metabolism upregulation has been associated with MYC proto-oncogene (*MYC*) overexpression in RCC, inducing glutamine transporter and glutaminase (*GLS*) upregulation [56], followed by elevated glutamate and α-ketoglutarate levels, and lipid accumulation in RCC tumors [56].

### 4.3. Role of Lactate in Tumor Microenvironment

A glycolytic phenotype and high lactate production are common features of RCC [38]. Owing to lactate efflux with co-transport of H^+^ through MCTs, cancer cells maintain the intracellular pH at physiological levels, whereas tumor microenvironment becomes acidic [57], which has been associated with increased tumor aggressiveness [58]. Accordingly, high lactate levels have been associated with poor prognosis, high risk for development of metastasis and high tumor recurrence in several solid tumors [59]. Furthermore, lactate plays an important role in tumor microenvironment by supporting tumor growth and proliferation, as well as increasing migration, invasion, sustained angiogenesis, immune escape and therapy resistance [60,61,62], constituting a tumor metabolic fuel [43,63]. Indeed, HIF-1α stabilization by lactate was associated with increased vascular endothelial growth factor (VEGF) and kinase insert domain receptor (VEGFR2) expression levels by tumor and endothelial cells, respectively [64]. Moreover, lactate induces interleukin 8 (IL-8) production by endothelial cells in HIF-1α-independent way [65]. Nonetheless, during long hypoxia periods, N-Myc downstream-regulated (NDRG3) protein expression in cancer cells promotes VEGF, IL-8 and platelet and endothelial cell adhesion molecule 1 (CD31) up-regulation [66].

Remarkably, lactate was shown to impact on immune surveillance, since high microenvironmental levels associated with decreased T cells cytotoxic and natural killer cells activity, along with reduced dendritic cells cytokine release and differentiation [62]. Additionally, lactate-induced HIF-1α stabilization stimulates polarization of tumor-associated macrophages, contributing to immunosuppression [67].

Other lines of evidence regarding lactate role in tumorigenesis include the positive correlation found between tumor lactate content and radioresistance in human solid tumors [68].

## 5. Epigenetic Mechanisms

**Figure 3:**
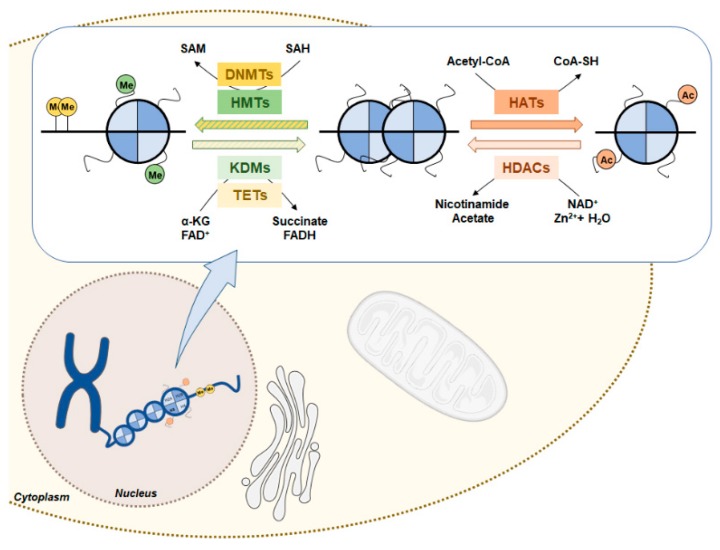
DNA methylation and histone PTM in gene expression regulation. DMNTs catalyze cytosine methylation using SAM as a methyl donor, which is converted to SAH. TETs revert DNA methylation by a hydroxylation α-KG-dependent reaction. Histone methylation patterns are established by HMTs using SAM as methyl donor. Inversely, histone demethylases KDMs remove the methyl group of histone tails using FAD^+^ or α-KG as cofactor. HATs imprint acetylation marks in histone transferring the acetyl group of acetyl-CoA to histone tails. Histone acetylation marks can be removed by HDACs using as co-factor NAD^+^ or Zn^2+^. Abbreviations: CoA-SH, Coenzyme A; DMNT, DNA methyltransferase; HAT, Histone acetyltransferase; HDAC, Histone deacetylase; NAD, Nicotinamide adenine dinucleotide; FAD, Flavin adenine dinucleotide; HMT, Histone methyltransferase; KDM, Histone lysine demethylase; SAH, S-adenosylhomocysteine; SAM, S-adenosylmethionine; TET, Ten-eleven translocation.

Epigenetics are heritable and reversible changes in gene expression patterns that result from alterations in chromatin and not in DNA sequence [69]. Epigenetic marks are crucial to maintain genomic stability, chromosome imprinting and cell differentiation in normal development [70]. Nevertheless, cellular epigenetic landscape plasticity is affected by genetic, environmental or metabolic stimuli allowing for cellular adaptation but that might lead to neoplastic transformation [71]. Epigenetic deregulation is a cancer hallmark, characterized by global gene expression deregulation with activation of proto-oncogenes and silencing of tumor suppressors genes due to changes in DNA methylation, histone post-translational modifications and chromatin remodeling complexes (Figure 3) [72,73].

### 5.1. DNA Methylation

DNA methylation is the most well studied epigenetic mechanism. In cancer, DNA methylation occurs mostly in cytosines of gene promoter cytosine-phosphate-guanine (CpG) islands, resulting in transcription repression [74]. DNA methyltransferases (DNMT1, DNMT3a and DNMT3b) are responsible for catalyzing the transfer of a methyl group from S-adenosyl-L-methionine (SAM) to 5′ carbon of cytosine ring, resulting in 5-methyl-cytosine (5mC) (Figure 3) [75]. DMNT1 preferentially binds to hemi-methylated sites, being responsible for DNA methylation patterns maintenance during replication [76]. Conversely, DNMT3a and DNMT3b are responsible for de novo methylation in unmethylated DNA duplex [77]. Nevertheless, DNA methylation process may be reverted by ten-eleven translocation (TET) proteins, which catalyze 5mC oxidation to 5hmC (5-hydroxymethyl-cytosine) in α-ketoglutarate-dependent manner (Figure 3) [78].

In normal cells, DNA methylation is associated with repetitive genomic regions, whereas gene promoters are generally unmethylated [79]. Contrarily, cancer cells display global DNA hypomethylation resulting in genome instability with proto-oncogenes activation, loss of imprinting and high mutation rates. Furthermore, aberrant gene promoter hypermethylation in cancer is associated with tumor suppressor genes’ transcription silencing [79,80].

### 5.2. Histone Post-Translational Modifications

Covalent modifications of histone tails regulate chromatin structure, controlling DNA transcription, replication and repair [81]. The most common post-translational modifications (PTMs) include methylation and acetylation of specific amino acid motifs of histones’ tails. These are controlled by histone methyltransferases (HMTs) or histone acetyltransferases (HATs), which establish the marks (writers), and by histone lysine demethylases (KDMs) or histone deacetylases (HDACs), which remove them (erasers) (Figure 3) [82].

Regarding histone methylation, HMTs catalyze the methylation reaction in lysine or arginine residues of histone tails using SAM as a methyl donor. To revert this condition, KDMs remove the methyl group from a methylated lysine by oxidation in a flavin adenine dinucleotide (FAD) or α-ketoglutarate (α-KG) dependent manner [83]. These enzymes have been found deregulated in several tumors. Histone methylation and consequent alteration of transcriptional status is dependent on the methylation degree and the residue modified. Trimethylation of H3K4 is found at transcriptionally active gene promoters, whereas H3K9me and H3K27me3 marks are associated with transcriptional repression [84]. Additionally, H4K20me3, a repressive histone mark, is also frequently found in human cancers [85].

Acetylation of histone lysine residues is established by HATs, using acetyl-CoA as acetyl donor [86]. The addition of acetyl group neutralizes the positive charges of histones decreasing DNA compaction, allowing protein binding and transcription activation [87]. Contrarily, deacetylation leads to DNA condensation by restoring the positive charges of lysine, with consequent transcription abrogation [87]. HDACs are responsible for removing acetyl groups and are categorized into four classes [88]. Classes I, II and IV share a catalytic mechanism in which zinc is used to catalyze hydrolysis of the lysine-amino bond [89]. Conversely, HDAC Class III, or Sirtuins (SIRTs), rely on NAD^+^ for deacetylation activity [90]. HATs and HDACs enzymes’ deregulation have been implicated in tumorigenesis and metastasis [91]. Indeed, loss of histone H4 Lys16 acetylation is defined as a common feature in cancer development [85]. Moreover, H3K9 hypoacetylation was associated with a high proliferative profile and higher recurrence rates in ependymal tumors [92].

### 5.3. Epigenetic Alterations in Renal Cell Carcinoma

**Figure 4:**
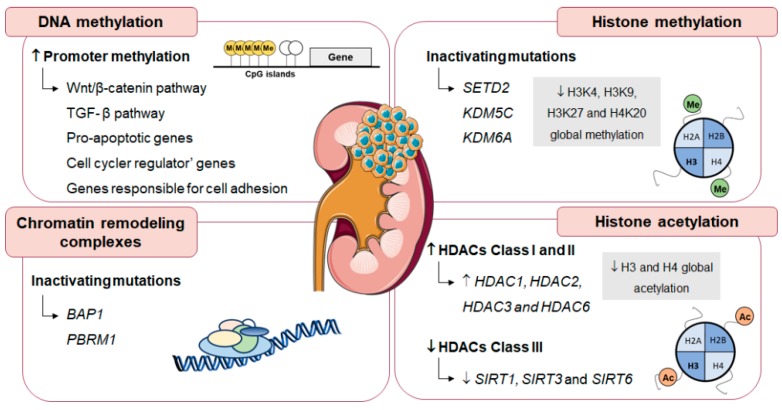
Epigenetic signature of RCC. Aberrant DNA methylation, chromatin remodeling complexes, and histone post-translational modifications regulate gene transcription and DNA stability through alterations in chromatin structure, contributing to RCC tumorigenesis and aggressiveness. Abbreviations: BAP1, BRCA1 associated protein 1; CpG, Cytosine-phosphate-guanine; PBRM1, Polybromo 1; SETD2, SET domain containing 2; SIRT, Sirtuin.

Epigenetic mechanisms allow cell adaptation to environmental changes, but also fueling cancer cells phenotype by supporting cell proliferation and metabolic reprogramming [93]. In RCC, alterations in DNA methylation profile and deregulation of genes involved in histone modifications and chromatin remodeling are frequently found (Figure 4) [94].

RCC commonly display high methylation levels at CpG islands associated with glycolytic phenotype, as well as aggressive behavior [11,21,95]. In RCC, epigenetic silencing through gene promoter hypermethylation often downregulates genes related with Wnt/β-catenin and transforming growth factor (TGF) β pathway, pro-apoptotic genes, cell cycle regulator’ genes and genes responsible for cell adhesion [96], implicated in cancer proliferation and metastization [97].

Regarding chromatin remodeling enzymes, ccRCC commonly harbor *BAP1* and *PBRM1* inactivating mutations (mutated in approximately 15% and 50% of ccRCC cases, respectively) [98]. BAP1-deficient ccRCC tumors are associated with poor prognosis [99], whereas loss of PBRM1 expression increases tumor aggressiveness [100]. Moreover, inactivating mutations in *SETD2*, *KDM5C* and *KMD6A* were also found in RCC (mutated in approximately 5–15% of ccRCC cases) [101]. As a result of HTMs and KDMs deregulation, RCC display a specific histone methylation profile. Indeed, lower global H3K4 methylation levels are associated with poor prognosis and higher recurrence rates in RCC [102,103]. H3K9 and H4K20 global methylation is downregulated in RCC and has been correlated with tumor aggressiveness [103,104]. Furthermore, lower H3K27 methylation levels were found in RCC compared to oncocytomas, associating with higher pathological stage and grade, as well as lymph node and distant metastasis [105].

Globally, HDACs Class I and II seem to act as oncogenes. Namely, HDAC2 overexpression was associated with high proliferation index [106] and high HDAC3 expression levels were found in RCC [107], although an inverse correlation was depicted between pathological stage and HDAC3 levels [106]. Moreover, in vitro assays associated HDAC1 and HDAC6 overexpression with cell invasion, migration and motility modulation, by increasing matrix metalloproteinase expression and decreasing acetylated α-tubulin expression [108]. Furthermore, a The Cancer Genome Atlas (TCGA) dataset analysis associated high HDAC1 mRNA levels with ccRCC worse overall survival [108]. Furthermore, high HDAC6 expression levels were displayed by high grade RCC and were shown to be an independent poor prognostic factor [109]. Paradoxically, although ccRCC samples depict lower HDAC9 levels than normal adjacent tissues, high HDAC9 expression was associated with poor prognosis [110]. Remarkably, global histone acetylation downregulation was found in RCC and associated with patients’ outcome [111]. Decreased H3 and H4 global acetylation was associated with high tumor grade, advanced stage, including distant metastasis, and tumor progression [111,112]. Furthermore, in ccRCC cell lines, H3 acetylation restoration inhibited cell proliferation, whereas apoptosis and cell cycle arrest were induced [112].

Regarding HDACs Class III, SIRT1, SIRT3 and SIRT6 were shown downregulated in RCC samples compared with normal kidney tissues, suggesting a putative role as onco-suppressors. Furthermore, in the same study, SIRT3 higher expression levels were correlated with better patients’ outcome [113]. SIRTs, in addition to chromatin structure regulation, have been suggested to modulate several cellular processes including metabolism and genomic stability maintenance [114]. Indeed, SIRT1, SIRT3 and SIRT6 downregulation was associated with HIF-1α-mediated glycolytic metabolism activation, tumor growth and glucose homeostasis [115,116,117].

## 6. Interplay Between Metabolism and Epigenetic Mechanisms

Cancer cells display several genetic and epigenetic alterations that enable metabolic adaptation and sustain cell proliferation and survival [118]. The cancer metabolic switch leads to metabolites accumulation, which have been suggested to modulate epigenetic factors and consequent gene expression. Indeed, epigenetic enzymes involved in DNA and histone modifications require intermediates of cellular metabolic pathways, such as acetyl-CoA, α-KG, SAM and NAD^+^ as cofactors or substrates [119,120]. Furthermore, metabolic pathways disruption can also lead to accumulation of metabolites such as coenzyme A (CoA-SH), S-adenosylhomocysteine (SAH), β-hydroxybutyrate (β-OHB), 2-hydroxyglutarate (2-HG), fumarate, succinate and lactate, which inhibit specific epigenetic players [121].

### 6.1. Renal Cell Carcinoma Metabolism and Histone Acetylation Modulation

As previously mentioned, RCC display high glycolytic metabolism with increased pyruvate conversion into lactate [45]. Although the effect of these two metabolites in epigenetic landscape has not yet been described for RCC, these two metabolites were shown to act as HDACs inhibitors in other tumors. Accordingly, increased tumor cell apoptosis was associated with HDACs inhibition by pyruvate, suggesting a negative role in tumor formation of the latter [122]. Additionally, most pyruvate is directly converted into lactate to keep the NAD^+^/NADH ratio, sustaining glycolysis required by cancer cells. Conversely, lactate inhibition of HDACs Classes I and II was reported for colon and cervical cancers. Lactate promotes histone acetylation and gene expression through HDAC-inhibition mechanism contributing to DNA repair and to anticancer therapy resistance [123,124]. Furthermore, increased histone acetylation levels have been found after exposing breast cancer cells to lactate, as well as consequent increased expression on stemness-related genes and an association between lactate induced genes and patient outcome. However, in that study, lactate-associated acetylation was justified not by HDACs inhibition but through HATs activation by acetyl-CoA pool increase [125]. Additionally, other metabolites, such as butyrate and β-OHB, are also well known HDACs inhibitors [126,127]. Interestingly, butyrate plays a dual role in cells’ epigenetic regulation. In cancer cells, Warburg effect leads to increased butyrate levels because it is not converted into acetyl-CoA. In these conditions, butyrate accumulation inhibits HDACs Class I, II and IV activity and cell proliferation by transcription of pro-apoptotic genes [128]. Instead, when cells use butyrate as fuel through acetyl-CoA production, proliferation is stimulated and cancer growth is facilitated [128]. Curiously, β-OHB seems to have an effect similar to butyrate. In absence of glycolytic metabolism, β-OHB is used as oxidative energy source instead of epigenetic regulator, inducing tumor growth [129]. In the presence of glycolytic metabolism, however, β-OHB is not converted into acetyl-CoA but it accumulates, leading to HDACs Class I and II inhibition [125]. Additionally, an in vivo study demonstrated specific inhibition of HDACs Class I by β-OHB with consequent increase in global histone acetylation, which was correlated with oxidative stress resistance, inducing transcriptional activity [130]. In parallel, cancer glycolysis decreases NAD^+^/NADH ratio, essential for SIRTs activity, as these enzymes depend on NAD^+^ as cofactor. Thus, SIRTs inhibition seems to contribute to hyperacetylation and aberrant gene transcription, leading to cancer progression [118]. Nevertheless, additional studies are needed to better understand the interactions between metabolic status and SIRTs activity in cancer.

In RCC, glycolytic metabolism induced by HIF-1α pathway activation leads to TCA cycle suppression by blocking pyruvate conversion into acetyl-CoA [131]. In this situation, tumor cells use other sources to obtain acetyl-CoA essential for lipid synthesis, as well as for HATs function [132]. Cancer cells can reprogram its metabolism to produce mitochondrial citrate, which is transported to cytosol and converted into acetyl-CoA through ATP-citrate lyase [133]. In these conditions, acetyl-CoA plays a role in histone acetylation in response to glucose and growth factor stimulation [134,135]. Cytosolic acetate is another acetyl-CoA source [133]. Under glucose restriction or hypoxic stress, acetate is used as an immediate metabolic precursor for fatty acid synthesis and also induces expression of genes involved in de novo lipid synthesis and cancer cell survival, by promoting histone acetylation at specific promoter regions [136]. Additionally, acetyl-CoA generated from glutamine was shown to be involved in histone acetylation, inducing the expression of genes involved in lipid metabolism [137].

**Figure 5:**
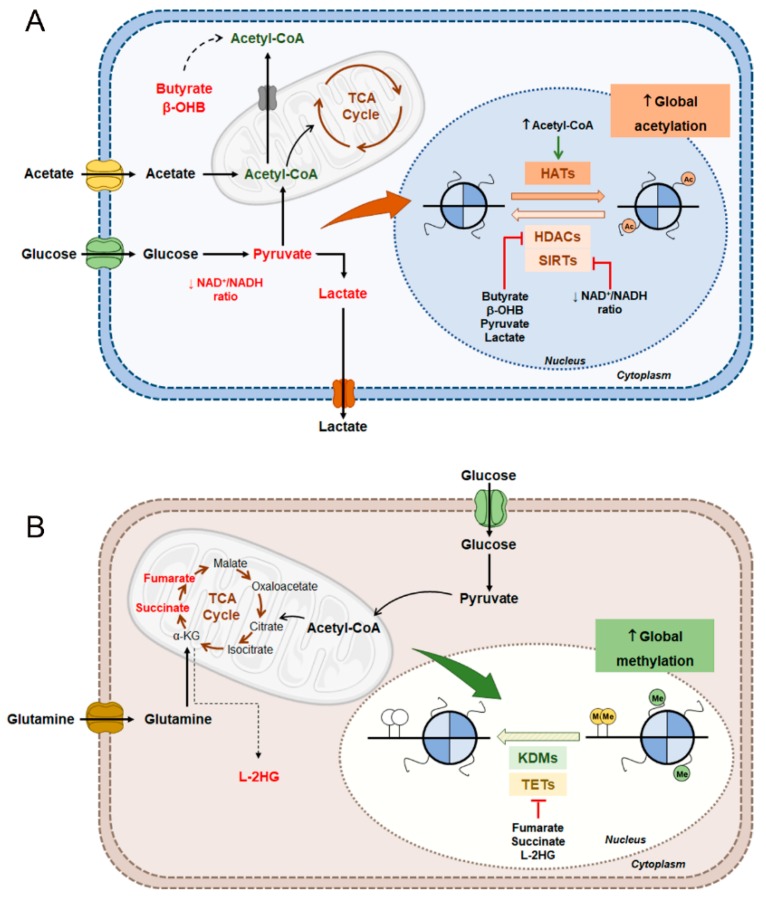
Putative metabolic reprogramming of epigenetic enzymes in RCC. Epigenetic mechanisms can be modulated by the available metabolites in the cell, contributing to aberrant gene expression. (**A**) Regarding histone acetylation, HATs activity is stimulated by high acetyl-CoA levels. In contrast, HDACs activity can be inhibited by butyrate, β-hydroxybutyrate, pyruvate and lactate, whereas SIRTs activity is regulated by NAD^+^/NADH ratio. (**B**) DNA and histone demethylation can be decreased by inhibition of KDMs or TETs due to presence of fumarate, succinate and L-2HG, metabolites with a similar structure to α-KG. Abbreviations: L-2HG, L enantiomer of 2-hydroxyglutarate; β-OHB, Beta-hydroxybutyrate.

Thus, taking in account the high glycolytic metabolism of RCC, these tumors seem to disclose global histone hyperacetylation and consequent aberrant gene transcription, possibly due to increased HATs activity induced by acetyl-CoA, and also HDACs inhibition by several metabolites that contribute to cancer progression and aggressiveness (Figure 5A) [118]. Further studies to clarify this hypothesis in this specific model are still needed.

### 6.2. Renal Cell Carcinoma Metabolism in DNA and Histone Methylation Modulation

Fumarate and succinate accumulation are typically found in FH and SDH-deficient RCC. Both metabolites have been related with α-KG-dependent histone inhibition and DNA demethylases, namely TETs and KDMs [138,139]. In tumors with *SDH* mutations, succinate accumulation associates with DNA hypermethylation phenotype [140]. Inhibition of histone and DNA demethylases was associated with increased tumor aggressiveness and invasion potential, mediated by epigenetic silencing of genes involved in cell differentiation and epithelial mesenchymal transition (EMT) [140,141]. Moreover, fumarate was also associated with tumor aggressiveness. Sciacovelli et al. demonstrated that intracellular fumarate accumulation drives changes in EMT genes, such as zinc finger E-box binding homeobox (*ZEB*), snail family transcriptional repressor 1 (*SNAI1*) and vimentin (*VIM*), through inhibition of TETs and consequent anti-metastatic microRNA cluster downregulation, in murine renal carcinoma cells [142].

More recently, L enantiomer of 2- hydroxyglutarate (L-2HG) was found to be accumulated in RCC [143]. This metabolite is produced from α-KG by LDHA under hypoxic conditions [144,145] and was demonstrated to be a potent TETs and KDMs inhibitor [146,147]. In RCC, increased L-2HG levels are mediated by L-2HG dehydrogenase (L2HGDH) reduced expression due to copy number loss [143]. Additionally, Shim et al. reported an inverse association between high L-2HG levels and reduced DNA 5hmC levels due to TET activity inhibition [143]. Contrarily, L2HGDH restoration in RCC cells was associated with L-2HG decrease and consequent DNA and histone demethylation by re-activation of TETs and KDMs activity. Moreover, L2HGDH re-expression was able to attenuate the malignant phenotype by inhibiting L-2HG oncogenic role in RCC cell lines [143]. Hence, owing to mutations in *FH*, *SDH* and *L2HGDH* genes, which lead to TET and KMDs inhibition, aberrant accumulation of oncometabolites occurs in RCC, stimulating tumor growth and aggressiveness (Figure 5B) [148,149].

## 7. Conclusions and Perspectives

Although the majority of RCCs are currently diagnosed at initial stages, about 20% of patients already harbor metastases at the time of diagnosis, decreasing the survival rate to less than 20% [150]. Despite advances in targeted therapies, nephrectomy remains the mainstay of RCC treatment [151]. Nevertheless, systemic therapy based on immune system modulators has traditionally been used in metastatic disease, although with modest impact in patient outcome [152]. Over the past decade, significant improvements have been made, and new targeted therapies, including antiangiogenic agents, mTOR and immune checkpoint inhibitors, have emerged as promising alternatives to current therapies [153,154]. Notwithstanding the progress in this field, RCC remains associated with high recurrence rates, and a sizeable proportion of patients with initially localized disease develop metastasis during follow-up [155,156]. The identification of new tumor-associated markers that might allow the design of new therapeutic strategies in management of RCC patients is thus crucial. Cell metabolism deregulation, particularly Warburg effect, plays a major role in RCC development, contributing to accumulation of metabolites that regulate epigenetic factors. Hence, the study and discovery of new metabolic target therapies that might be used in combination with standard therapy might be of clinical value. Furthermore, the usage of drugs that reestablish defects in epigenetic machinery caused by metabolites’ accumulation may be a strategy deserving to be explored.

## References

[1] Bray F., Ferlay J., Soerjomataram I., Siegel R.L., Torre L.A., Jemal A. (2018). Global cancer statistics 2018: GLOBOCAN estimates of incidence and mortality worldwide for 36 cancers in 185 countries. CA Cancer J. Clin..

[2] Ljungberg B., Campbell S.C., Choi H.Y., Jacqmin D., Lee J.E., Weikert S., Kiemeney L.A. (2011). The epidemiology of renal cell carcinoma. Eur. Urol..

[3] Dhote R., Thiounn N., Debre B., Vidal-Trecan G. (2004). Risk factors for adult renal cell carcinoma. Urol. Clin. North Am..

[4] Qayyum T., Oades G., Horgan P., Aitchison M., Edwards J. (2012). The epidemiology and risk factors for renal cancer. Curr. Urol..

[5] Lucca I., Klatte T., Fajkovic H., de Martino M., Shariat S.F. (2015). Gender differences in incidence and outcomes of urothelial and kidney cancer. Nat. Rev. Urol..

[6] Clague J., Lin J., Cassidy A., Matin S., Tannir N.M., Tamboli P., Wood C.G., Wu X. (2009). Family history and risk of renal cell carcinoma: Results from a case-control study and systematic meta-analysis. Cancer Epidemiol. Biomark. Prev..

[7] Escudier B., Porta C., Schmidinger M., Rioux-Leclercq N., Bex A., Khoo V., Gruenvald V., Horwich A., Committee E.G. (2016). Renal cell carcinoma: ESMO Clinical Practice Guidelines for diagnosis, treatment and follow-up. Ann. Oncol..

[8] Muglia V.F., Prando A. (2015). Renal cell carcinoma: Histological classification and correlation with imaging findings. Radiol. Bras..

[9] Ricketts C.J., Linehan W.M. (2014). Intratumoral heterogeneity in kidney cancer. Nat. Genet..

[10] Haas N.B., Nathanson K.L. (2014). Hereditary kidney cancer syndromes. Adv. Chronic Kidney Dis..

[11] The Cancer Genome Atlas Research Network (2013). Comprehensive molecular characterization of clear cell renal cell carcinoma. Nature.

[12] Banks R.E., Tirukonda P., Taylor C., Hornigold N., Astuti D., Cohen D., Maher E.R., Stanley A.J., Harnden P., Joyce A. (2006). Genetic and epigenetic analysis of von Hippel-Lindau (VHL) gene alterations and relationship with clinical variables in sporadic renal cancer. Cancer Res..

[13] Ivan M., Kondo K., Yang H., Kim W., Valiando J., Ohh M., Salic A., Asara J.M., Lane W.S., Kaelin W.G. (2001). HIFalpha targeted for VHL-mediated destruction by proline hydroxylation: Implications for O_2_ sensing. Science.

[14] Semenza G.L. (2013). HIF-1 mediates metabolic responses to intratumoral hypoxia and oncogenic mutations. J. Clin. Investig..

[15] Gossage L., Eisen T., Maher E.R. (2015). VHL, the story of a tumour suppressor gene. Nat. Rev. Cancer.

[16] Maxwell P.H., Wiesener M.S., Chang G.W., Clifford S.C., Vaux E.C., Cockman M.E., Wykoff C.C., Pugh C.W., Maher E.R., Ratcliffe P.J. (1999). The tumour suppressor protein VHL targets hypoxia-inducible factors for oxygen-dependent proteolysis. Nature.

[17] Dengler V.L., Galbraith M., Espinosa J.M. (2014). Transcriptional regulation by hypoxia inducible factors. Crit. Rev. Biochem. Mol. Biol..

[18] Shen C., Kaelin W.G. (2013). The VHL/HIF axis in clear cell renal carcinoma. Semin. Cancer Biol..

[19] Schodel J., Grampp S., Maher E.R., Moch H., Ratcliffe P.J., Russo P., Mole D.R. (2016). Hypoxia, Hypoxia-inducible Transcription Factors, and Renal Cancer. Eur. Urol..

[20] Courthod G., Tucci M., Di Maio M., Scagliotti G.V. (2015). Papillary renal cell carcinoma: A review of the current therapeutic landscape. Crit. Rev. Oncol. Hematol..

[21] Linehan W.M., Spellman P.T., Ricketts C.J., Creighton C.J., Fei S.S., Davis C., Wheeler D.A., Murray B.A., Schmidt L., The Cancer Genome Atlas Research Network (2016). Comprehensive Molecular Characterization of Papillary Renal-Cell Carcinoma. N. Engl. J. Med..

[22] Delahunt B., Eble J.N. (1997). Papillary renal cell carcinoma: A clinicopathologic and immunohistochemical study of 105 tumors. Mod. Pathol..

[23] Albiges L., Guegan J., Le Formal A., Verkarre V., Rioux-Leclercq N., Sibony M., Bernhard J.C., Camparo P., Merabet Z., Molinie V. (2014). MET is a potential target across all papillary renal cell carcinomas: Result from a large molecular study of pRCC with CGH array and matching gene expression array. Clin. Cancer Res..

[24] Lubensky I.A., Schmidt L., Zhuang Z., Weirich G., Pack S., Zambrano N., Walther M.M., Choyke P., Linehan W.M., Zbar B. (1999). Hereditary and sporadic papillary renal carcinomas with c-met mutations share a distinct morphological phenotype. Am. J. Pathol..

[25] Schmidt L., Duh F.M., Chen F., Kishida T., Glenn G., Choyke P., Scherer S.W., Zhuang Z., Lubensky I., Dean M. (1997). Germline and somatic mutations in the tyrosine kinase domain of the MET proto-oncogene in papillary renal carcinomas. Nat. Genet..

[26] Haas N.B., Nathanson K.L. (2014). Hereditary Renal Cancer Syndromes. Adv. Chronic Kidney Dis..

[27] Peruzzi B., Bottaro D.P. (2006). Targeting the c-Met signaling pathway in cancer. Clin. Cancer Res..

[28] Farber L.J., Furge K., Teh B.T. (2012). Renal cell carcinoma deep sequencing: Recent developments. Curr. Oncol. Rep..

[29] Tomlinson I.P., Alam N.A., Rowan A.J., Barclay E., Jaeger E.E., Kelsell D., Leigh I., Gorman P., Lamlum H., Rahman S. (2002). Germline mutations in FH predispose to dominantly inherited uterine fibroids, skin leiomyomata and papillary renal cell cancer. Nat. Genet..

[30] Isaacs J.S., Jung Y.J., Mole D.R., Lee S., Torres-Cabala C., Chung Y.L., Merino M., Trepel J., Zbar B., Toro J. (2005). HIF overexpression correlates with biallelic loss of fumarate hydratase in renal cancer: Novel role of fumarate in regulation of HIF stability. Cancer Cell.

[31] Yang Y., Lane A.N., Ricketts C.J., Sourbier C., Wei M.H., Shuch B., Pike L., Wu M., Rouault T.A., Boros L.G. (2013). Metabolic reprogramming for producing energy and reducing power in fumarate hydratase null cells from hereditary leiomyomatosis renal cell carcinoma. PLoS ONE.

[32] Hanahan D., Weinberg R.A. (2011). Hallmarks of cancer: The next generation. Cell.

[33] DeBerardinis R.J., Lum J.J., Hatzivassiliou G., Thompson C.B. (2008). The biology of cancer: Metabolic reprogramming fuels cell growth and proliferation. Cell Metab..

[34] Vander Heiden M.G., Cantley L.C., Thompson C.B. (2009). Understanding the Warburg effect: The metabolic requirements of cell proliferation. Science.

[35] House S.W., Warburg O., Burk D., Schade A.L. (1956). On respiratory impairment in cancer cells. Science.

[36] Warburg O. (1956). On the origin of cancer cells. Science.

[37] Zheng J. (2012). Energy metabolism of cancer: Glycolysis versus oxidative phosphorylation (Review). Oncol. Lett..

[38] Gatenby R.A., Gillies R.J. (2004). Why do cancers have high aerobic glycolysis?. Nat. Rev. Cancer.

[39] Gillies R.J., Robey I., Gatenby R.A. (2008). Causes and consequences of increased glucose metabolism of cancers. J. Nucl. Med..

[40] Pavlova N.N., Thompson C.B. (2016). The emerging hallmarks of cancer metabolism. Cell Metab..

[41] Liberti M.V., Locasale J.W. (2016). The Warburg Effect: How Does it Benefit Cancer Cells?. Trends Biochem. Sci..

[42] Feron O. (2009). Pyruvate into lactate and back: From the Warburg effect to symbiotic energy fuel exchange in cancer cells. Radiother. Oncol..

[43] Semenza G.L. (2008). Tumor metabolism: Cancer cells give and take lactate. J. Clin. Investig..

[44] Linehan W.M., Srinivasan R., Schmidt L.S. (2010). The genetic basis of kidney cancer: A metabolic disease. Nat. Rev. Urol..

[45] Sudarshan S., Karam J.A., Brugarolas J., Thompson R.H., Uzzo R., Rini B., Margulis V., Patard J.J., Escudier B., Linehan W.M. (2013). Metabolism of kidney cancer: From the lab to clinical practice. Eur. Urol..

[46] Semenza G.L. (2007). HIF-1 mediates the Warburg effect in clear cell renal carcinoma. J. Bioenerg. Biomembr..

[47] Masoud G.N., Li W. (2015). HIF-1α pathway: Role, regulation and intervention for cancer therapy. Acta Pharm. Sin. B.

[48] Soltysova A., Breza J., Takacova M., Feruszova J., Hudecova S., Novotna B., Rozborilova E., Pastorekova S., Kadasi L., Krizanova O. (2015). Deregulation of energetic metabolism in the clear cell renal cell carcinoma: A multiple pathway analysis based on microarray profiling. Int. J. Oncol..

[49] Sanders E., Diehl S. (2015). Analysis and interpretation of transcriptomic data obtained from extended Warburg effect genes in patients with clear cell renal cell carcinoma. Oncoscience.

[50] Lim H.Y., Yip Y.M., Chiong E., Tiong H.Y., Halliwell B., Esuvaranathan K., Wong K.P. (2015). Metabolic signatures of renal cell carcinoma. Biochem. Biophys. Res. Commun..

[51] Van der Mijn J.C., Panka D.J., Geissler A.K., Verheul H.M., Mier J.W. (2016). Novel drugs that target the metabolic reprogramming in renal cell cancer. Cancer Metab..

[52] Wettersten H.I., Aboud O.A., Lara P.N., Weiss R.H. (2017). Metabolic reprogramming in clear cell renal cell carcinoma. Nat. Rev. Nephrol..

[53] Pinthus J.H., Whelan K.F., Gallino D., Lu J.P., Rothschild N. (2011). Metabolic features of clear-cell renal cell carcinoma: Mechanisms and clinical implications. Can. Urol. Assoc. J..

[54] Langbein S., Frederiks W.M., zur Hausen A., Popa J., Lehmann J., Weiss C., Alken P., Coy J.F. (2008). Metastasis is promoted by a bioenergetic switch: New targets for progressive renal cell cancer. Int. J. Cancer.

[55] Horiguchi A., Asano T., Asano T., Ito K., Sumitomo M., Hayakawa M. (2008). Fatty acid synthase over expression is an indicator of tumor aggressiveness and poor prognosis in renal cell carcinoma. J. Urol..

[56] Shroff E.H., Eberlin L.S., Dang V.M., Gouw A.M., Gabay M., Adam S.J., Bellovin D.I., Tran P.T., Philbrick W.M., Garcia-Ocana A. (2015). MYC oncogene overexpression drives renal cell carcinoma in a mouse model through glutamine metabolism. Proc. Natl. Acad. Sci. USA.

[57] Neri D., Supuran C.T. (2011). Interfering with pH regulation in tumours as a therapeutic strategy. Nat. Rev. Drug Discov..

[58] Miranda-Goncalves V., Reis R.M., Baltazar F. (2016). Lactate Transporters and pH Regulation: Potential Therapeutic Targets in Glioblastomas. Curr. Cancer Drug Targets.

[59] Walenta S., Mueller-Klieser W.F. (2004). Lactate: Mirror and motor of tumor malignancy. Semin. Radiat. Oncol..

[60] Hirschhaeuser F., Sattler U.G., Mueller-Klieser W. (2011). Lactate: A metabolic key player in cancer. Cancer Res..

[61] Marchiq I., Pouyssegur J. (2016). Hypoxia, cancer metabolism and the therapeutic benefit of targeting lactate/H(+) symporters. J. Mol. Med. (Berl.).

[62] Romero-Garcia S., Moreno-Altamirano M.M., Prado-Garcia H., Sanchez-Garcia F.J. (2016). Lactate Contribution to the Tumor Microenvironment: Mechanisms, Effects on Immune Cells and Therapeutic Relevance. Front. Immunol..

[63] Draoui N., Feron O. (2011). Lactate shuttles at a glance: From physiological paradigms to anti-cancer treatments. Dis. Model. Mech..

[64] Sonveaux P., Copetti T., De Saedeleer C.J., Vegran F., Verrax J., Kennedy K.M., Moon E.J., Dhup S., Danhier P., Frerart F. (2012). Targeting the lactate transporter MCT1 in endothelial cells inhibits lactate-induced HIF-1 activation and tumor angiogenesis. PLoS ONE.

[65] Vegran F., Boidot R., Michiels C., Sonveaux P., Feron O. (2011). Lactate influx through the endothelial cell monocarboxylate transporter MCT1 supports an NF-kappaB/IL-8 pathway that drives tumor angiogenesis. Cancer Res..

[66] Lee D.C., Sohn H.A., Park Z.Y., Oh S., Kang Y.K., Lee K.M., Kang M., Jang Y.J., Yang S.J., Hong Y.K. (2015). A lactate-induced response to hypoxia. Cell.

[67] Colegio O.R., Chu N.Q., Szabo A.L., Chu T., Rhebergen A.M., Jairam V., Cyrus N., Brokowski C.E., Eisenbarth S.C., Phillips G.M. (2014). Functional polarization of tumour-associated macrophages by tumour-derived lactic acid. Nature.

[68] Sattler U.G., Meyer S.S., Quennet V., Hoerner C., Knoerzer H., Fabian C., Yaromina A., Zips D., Walenta S., Baumann M. (2010). Glycolytic metabolism and tumour response to fractionated irradiation. Radiother. Oncol..

[69] Allis C.D., Jenuwein T. (2016). The molecular hallmarks of epigenetic control. Nat. Rev. Genet..

[70] Cantone I., Fisher A.G. (2013). Epigenetic programming and reprogramming during development. Nat. Struct. Mol. Biol..

[71] Flavahan W.A., Gaskell E., Bernstein B.E. (2017). Epigenetic plasticity and the hallmarks of cancer. Science.

[72] Brettingham-Moore K.H., Taberlay P.C., Egger G., Arimondo P. (2015). Cancer Epigenetics. Drug Discovery in Cancer Epigenetics.

[73] Egger G., Liang G., Aparicio A., Jones P.A. (2004). Epigenetics in human disease and prospects for epigenetic therapy. Nature.

[74] Baylin S.B. (2005). DNA methylation and gene silencing in cancer. Nat. Clin. Pract. Oncol..

[75] Chen Z.X., Riggs A.D. (2011). DNA methylation and demethylation in mammals. J Biol. Chem..

[76] Li E., Bestor T.H., Jaenisch R. (1992). Targeted mutation of the DNA methyltransferase gene results in embryonic lethality. Cell.

[77] Okano M., Bell D.W., Haber D.A., Li E. (1999). DNA methyltransferases Dnmt3a and Dnmt3b are essential for de novo methylation and mammalian development. Cell.

[78] Ito S., Shen L., Dai Q., Wu S.C., Collins L.B., Swenberg J.A., He C., Zhang Y. (2011). Tet proteins can convert 5-methylcytosine to 5-formylcytosine and 5-carboxylcytosine. Science.

[79] Kulis M., Esteller M., Herceg Z., Ushijima T. (2010). DNA methylation and cancer. Advances in Genetics.

[80] Robertson K.D. (2005). DNA methylation and human disease. Nat. Rev. Genet..

[81] Kouzarides T. (2007). Chromatin modifications and their function. Cell.

[82] Vaquero A., Loyola A., Reinberg D. (2003). The constantly changing face of chromatin. Sci. Aging Knowl. Environ..

[83] Song Y., Wu F., Wu J. (2016). Targeting histone methylation for cancer therapy: Enzymes, inhibitors, biological activity and perspectives. J. Hematol. Oncol..

[84] Li B., Carey M., Workman J.L. (2007). The role of chromatin during transcription. Cell.

[85] Fraga M.F., Ballestar E., Villar-Garea A., Boix-Chornet M., Espada J., Schotta G., Bonaldi T., Haydon C., Ropero S., Petrie K. (2005). Loss of acetylation at Lys16 and trimethylation at Lys20 of histone H4 is a common hallmark of human cancer. Nat. Genet..

[86] Roth S.Y., Denu J.M., Allis C.D. (2001). Histone acetyltransferases. Annu. Rev. Biochem..

[87] Jenuwein T., Allis C.D. (2001). Translating the histone code. Science.

[88] Seto E., Yoshida M. (2014). Erasers of histone acetylation: The histone deacetylase enzymes. Cold Spring Harb. Perspect. Biol..

[89] Zhang L., Zhang J., Jiang Q., Zhang L., Song W. (2018). Zinc binding groups for histone deacetylase inhibitors. J. Enzyme Inhib. Med. Chem..

[90] Imai S., Armstrong C.M., Kaeberlein M., Guarente L. (2000). Transcriptional silencing and longevity protein Sir2 is an NAD-dependent histone deacetylase. Nature.

[91] Cohen I., Poreba E., Kamieniarz K., Schneider R. (2011). Histone modifiers in cancer: Friends or foes?. Genes Cancer.

[92] Ebrahimi A., Schittenhelm J., Honegger J., Schluesener H. (2013). Prognostic relevance of global histone 3 lysine 9 acetylation in ependymal tumors. J. Neurosurg..

[93] Feil R., Fraga M.F. (2012). Epigenetics and the environment: Emerging patterns and implications. Nat. Rev. Genet..

[94] Morris M.R., Latif F. (2017). The epigenetic landscape of renal cancer. Nat. Rev. Nephrol..

[95] Malouf G., Zhang J., Tannir N.M., Thompson E., Spano J.-P., Khayat D., Su X. (2014). Association of CpG island methylator phenotype with clear-cell renal cell carcinoma aggressiveness. J. Clin. Oncol..

[96] Shenoy N., Vallumsetla N., Zou Y., Galeas J.N., Shrivastava M., Hu C., Susztak K., Verma A. (2015). Role of DNA methylation in renal cell carcinoma. J. Hematol. Oncol..

[97] Hu C.Y., Mohtat D., Yu Y., Ko Y.A., Shenoy N., Bhattacharya S., Izquierdo M.C., Park A.S., Giricz O., Vellamkutla N. (2014). Kidney cancer is characterized by aberrant methylation of tissue-specific enhancers that are prognostic for overall survival. Clin. Cancer Res..

[98] Brugarolas J. (2013). PBRM1 and BAP1 as novel targets for renal cell carcinoma. Cancer J..

[99] Kapur P., Pena-Llopis S., Christie A., Zhrebker L., Pavia-Jimenez A., Rathmell W.K., Xie X.J., Brugarolas J. (2013). Effects on survival of BAP1 and PBRM1 mutations in sporadic clear-cell renal-cell carcinoma: A retrospective analysis with independent validation. Lancet Oncol..

[100] Pawlowski R., Muhl S.M., Sulser T., Krek W., Moch H., Schraml P. (2013). Loss of PBRM1 expression is associated with renal cell carcinoma progression. Int. J. Cancer.

[101] Chi P., Allis C.D., Wang G.G. (2010). Covalent histone modifications—Miswritten, misinterpreted and mis-erased in human cancers. Nat. Rev. Cancer.

[102] Ellinger J., Kahl P., Mertens C., Rogenhofer S., Hauser S., Hartmann W., Bastian P.J., Buttner R., Muller S.C., von Ruecker A. (2010). Prognostic relevance of global histone H3 lysine 4 (H3K4) methylation in renal cell carcinoma. Int. J. Cancer.

[103] Seligson D.B., Horvath S., McBrian M.A., Mah V., Yu H., Tze S., Wang Q., Chia D., Goodglick L., Kurdistani S.K. (2009). Global levels of histone modifications predict prognosis in different cancers. Am. J. Pathol..

[104] Rogenhofer S., Kahl P., Holzapfel S., Von Ruecker A., Mueller S.C., Ellinger J. (2012). Decreased levels of histone H3K9me1 indicate poor prognosis in patients with renal cell carcinoma. Anticancer Res..

[105] Rogenhofer S., Kahl P., Mertens C., Hauser S., Hartmann W., Buttner R., Muller S.C., von Ruecker A., Ellinger J. (2012). Global histone H3 lysine 27 (H3K27) methylation levels and their prognostic relevance in renal cell carcinoma. BJU Int..

[106] Fritzsche F.R., Weichert W., Roske A., Gekeler V., Beckers T., Stephan C., Jung K., Scholman K., Denkert C., Dietel M. (2008). Class I histone deacetylases 1, 2 and 3 are highly expressed in renal cell cancer. BMC Cancer.

[107] Cha T.L., Chuang M.J., Wu S.T., Sun G.H., Chang S.Y., Yu D.S., Huang S.M., Huan S.K., Cheng T.C., Chen T.T. (2009). Dual degradation of aurora A and B kinases by the histone deacetylase inhibitor LBH589 induces G2-M arrest and apoptosis of renal cancer cells. Clin. Cancer Res..

[108] Ramakrishnan S., Ku S., Ciamporcero E., Miles K.M., Attwood K., Chintala S., Shen L., Ellis L., Sotomayor P., Swetzig W. (2016). HDAC 1 and 6 modulate cell invasion and migration in clear cell renal cell carcinoma. BMC Cancer.

[109] Zhang Z., Cao Y., Zhao W., Guo L., Liu W. (2017). HDAC6 serves as a biomarker for the prognosis of patients with renal cell carcinoma. Cancer Biomark..

[110] Liang Z., Mu X., Liang X., Hu K., Chen M. (2017). HDAC9 associates with distant metastasis and predicts poor prognosis in clear cell renal cell cancer. Int. J. Clin. Exp. Pathol..

[111] Mosashvilli D., Kahl P., Mertens C., Holzapfel S., Rogenhofer S., Hauser S., Buttner R., Von Ruecker A., Muller S.C., Ellinger J. (2010). Global histone acetylation levels: Prognostic relevance in patients with renal cell carcinoma. Cancer Sci..

[112] Kanao K., Mikami S., Mizuno R., Shinojima T., Murai M., Oya M. (2008). Decreased acetylation of histone H3 in renal cell carcinoma: A potential target of histone deacetylase inhibitors. J. Urol..

[113] Jeh S.U., Park J.J., Lee J.S., Kim D.C., Do J., Lee S.W., Choi S.M., Hyun J.S., Seo D.H., Lee C. (2017). Differential expression of the sirtuin family in renal cell carcinoma: Aspects of carcinogenesis and prognostic significance. Urol. Oncol..

[114] Chalkiadaki A., Guarente L. (2015). The multifaceted functions of sirtuins in cancer. Nat. Rev. Cancer.

[115] Lim J.H., Lee Y.M., Chun Y.S., Chen J., Kim J.E., Park J.W. (2010). Sirtuin 1 modulates cellular responses to hypoxia by deacetylating hypoxia-inducible factor 1alpha. Mol. Cell.

[116] Zhong L., D’Urso A., Toiber D., Sebastian C., Henry R.E., Vadysirisack D.D., Guimaraes A., Marinelli B., Wikstrom J.D., Nir T. (2010). The histone deacetylase Sirt6 regulates glucose homeostasis via Hif1alpha. Cell.

[117] Bell E.L., Emerling B.M., Ricoult S.J., Guarente L. (2011). SirT3 suppresses hypoxia inducible factor 1alpha and tumor growth by inhibiting mitochondrial ROS production. Oncogene.

[118] Wong C.C., Qian Y., Yu J. (2017). Interplay between epigenetics and metabolism in oncogenesis: Mechanisms and therapeutic approaches. Oncogene.

[119] Miranda-Gonçalves V., Lameirinhas A., Henrique R., Jeronimo C. (2018). Metabolism and epigenetic interplay in cancer: Regulation and putative therapeutic targets. Front. Genet..

[120] Rabhi N., Hannou S.A., Froguel P., Annicotte J.-S. (2017). Cofactors as metabolic sensors driving cell adaptation in physiology and disease. Front. Endocrinol..

[121] Kinnaird A., Zhao S., Wellen K.E., Michelakis E.D. (2016). Metabolic control of epigenetics in cancer. Nat. Rev. Cancer.

[122] Thangaraju M., Gopal E., Martin P.M., Ananth S., Smith S.B., Prasad P.D., Sterneck E., Ganapathy V. (2006). SLC5A8 triggers tumor cell apoptosis through pyruvate-dependent inhibition of histone deacetylases. Cancer Res..

[123] Latham T., Mackay L., Sproul D., Karim M., Culley J., Harrison D.J., Hayward L., Langridge-Smith P., Gilbert N., Ramsahoye B.H. (2012). Lactate, a product of glycolytic metabolism, inhibits histone deacetylase activity and promotes changes in gene expression. Nucleic Acids Res..

[124] Wagner W., Ciszewski W.M., Kania K.D. (2015). L- and D-lactate enhance DNA repair and modulate the resistance of cervical carcinoma cells to anticancer drugs via histone deacetylase inhibition and hydroxycarboxylic acid receptor 1 activation. Cell Commun. Signal..

[125] Martinez-Outschoorn U.E., Prisco M., Ertel A., Tsirigos A., Lin Z., Pavlides S., Wang C., Flomenberg N., Knudsen E.S., Howell A. (2011). Ketones and lactate increase cancer cell “stemness”, driving recurrence, metastasis and poor clinical outcome in breast cancer: Achieving personalized medicine via Metabolo-Genomics. Cell Cycle.

[126] Davie J.R. (2003). Inhibition of histone deacetylase activity by butyrate. J. Nutr..

[127] Newman J.C., Verdin E. (2014). β-hydroxybutyrate: Much more than a metabolite. Diabetes Res. Clin. Pract..

[128] Donohoe D.R., Collins L.B., Wali A., Bigler R., Sun W., Bultman S.J. (2012). The Warburg effect dictates the mechanism of butyrate-mediated histone acetylation and cell proliferation. Mol. Cell.

[129] Rodrigues L.M., Uribe-Lewis S., Madhu B., Honess D.J., Stubbs M., Griffiths J.R. (2017). The action of beta-hydroxybutyrate on the growth, metabolism and global histone H3 acetylation of spontaneous mouse mammary tumours: Evidence of a beta-hydroxybutyrate paradox. Cancer Metab..

[130] Shimazu T., Hirschey M.D., Newman J., He W., Shirakawa K., Le Moan N., Grueter C.A., Lim H., Saunders L.R., Stevens R.D. (2013). Suppression of oxidative stress by beta-hydroxybutyrate, an endogenous histone deacetylase inhibitor. Science.

[131] Huang D., Li C., Zhang H. (2014). Hypoxia and cancer cell metabolism. Acta Biochim. Biophys. Sin. (Shanghai).

[132] Pietrocola F., Galluzzi L., Bravo-San Pedro J.M., Madeo F., Kroemer G. (2015). Acetyl coenzyme A: A central metabolite and second messenger. Cell Metab..

[133] Kim J.A., Yeom Y.I. (2018). Metabolic Signaling to Epigenetic Alterations in Cancer. Biomol. Ther. (Seoul).

[134] Wellen K.E., Hatzivassiliou G., Sachdeva U.M., Bui T.V., Cross J.R., Thompson C.B. (2009). ATP-citrate lyase links cellular metabolism to histone acetylation. Science.

[135] Lee J.V., Carrer A., Shah S., Snyder N.W., Wei S., Venneti S., Worth A.J., Yuan Z.F., Lim H.W., Liu S. (2014). Akt-dependent metabolic reprogramming regulates tumor cell histone acetylation. Cell Metab..

[136] Gao X., Lin S.H., Ren F., Li J.T., Chen J.J., Yao C.B., Yang H.B., Jiang S.X., Yan G.Q., Wang D. (2016). Acetate functions as an epigenetic metabolite to promote lipid synthesis under hypoxia. Nat. Commun..

[137] McDonnell E., Crown S.B., Fox D.B., Kitir B., Ilkayeva O.R., Olsen C.A., Grimsrud P.A., Hirschey M.D. (2016). Lipids Reprogram Metabolism to Become a Major Carbon Source for Histone Acetylation. Cell Rep..

[138] Xiao M., Yang H., Xu W., Ma S., Lin H., Zhu H., Liu L., Liu Y., Yang C., Xu Y. (2012). Inhibition of alpha-KG-dependent histone and DNA demethylases by fumarate and succinate that are accumulated in mutations of FH and SDH tumor suppressors. Genes Dev..

[139] Laukka T., Mariani C.J., Ihantola T., Cao J.Z., Hokkanen J., Kaelin W.G., Godley L.A., Koivunen P. (2016). Fumarate and Succinate Regulate Expression of Hypoxia-inducible Genes via TET Enzymes. J. Biol. Chem..

[140] Letouze E., Martinelli C., Loriot C., Burnichon N., Abermil N., Ottolenghi C., Janin M., Menara M., Nguyen A.T., Benit P. (2013). SDH mutations establish a hypermethylator phenotype in paraganglioma. Cancer Cell.

[141] Loriot C., Domingues M., Berger A., Menara M., Ruel M., Morin A., Castro-Vega L.J., Letouze E., Martinelli C., Bemelmans A.P. (2015). Deciphering the molecular basis of invasiveness in Sdhb-deficient cells. Oncotarget.

[142] Sciacovelli M., Goncalves E., Johnson T.I., Zecchini V.R., da Costa A.S., Gaude E., Drubbel A.V., Theobald S.J., Abbo S.R., Tran M.G. (2016). Fumarate is an epigenetic modifier that elicits epithelial-to-mesenchymal transition. Nature.

[143] Shim E.H., Livi C.B., Rakheja D., Tan J., Benson D., Parekh V., Kho E.Y., Ghosh A.P., Kirkman R., Velu S. (2014). L-2-Hydroxyglutarate: An epigenetic modifier and putative oncometabolite in renal cancer. Cancer Discov..

[144] Oldham W.M., Clish C.B., Yang Y., Loscalzo J. (2015). Hypoxia-Mediated Increases in L-2-hydroxyglutarate Coordinate the Metabolic Response to Reductive Stress. Cell Metab..

[145] Intlekofer A.M., Dematteo R.G., Venneti S., Finley L.W., Lu C., Judkins A.R., Rustenburg A.S., Grinaway P.B., Chodera J.D., Cross J.R. (2015). Hypoxia Induces Production of L-2-Hydroxyglutarate. Cell Metab..

[146] Xu W., Yang H., Liu Y., Yang Y., Wang P., Kim S.H., Ito S., Yang C., Wang P., Xiao M.T. (2011). Oncometabolite 2-hydroxyglutarate is a competitive inhibitor of alpha-ketoglutarate-dependent dioxygenases. Cancer Cell.

[147] Chowdhury R., Yeoh K.K., Tian Y.M., Hillringhaus L., Bagg E.A., Rose N.R., Leung I.K., Li X.S., Woon E.C., Yang M. (2011). The oncometabolite 2-hydroxyglutarate inhibits histone lysine demethylases. EMBO Rep..

[148] Sciacovelli M., Frezza C. (2016). Oncometabolites: Unconventional triggers of oncogenic signalling cascades. Free Radic. Biol. Med..

[149] Shim E.H., Sudarshan S. (2015). Another small molecule in the oncometabolite mix: L-2-Hydroxyglutarate in kidney cancer. Oncoscience.

[150] Sunela K.L., Kataja M.J., Lehtinen E.T., Salminen T.K., Kujala P.M., Virman J.P., Kellokumpu-Lehtinen P.L. (2009). Prognostic factors and long-term survival in renal cell cancer patients. Scand. J. Urol. Nephrol..

[151] Krabbe L.M., Bagrodia A., Margulis V., Wood C.G. (2014). Surgical management of renal cell carcinoma. Semin. Intervent. Radiol..

[152] Hutson T.E., Quinn D.I. (2005). Cytokine therapy: A standard of care for metastatic renal cell carcinoma?. Clin. Genitourin. Cancer.

[153] Erman M., Benekli M., Basaran M., Bavbek S., Buyukberber S., Coskun U., Demir G., Karabulut B., Oksuzoglu B., Ozkan M. (2016). Renal cell cancer: Overview of the current therapeutic landscape. Expert Rev. Anticancer Ther..

[154] Hutson T.E. (2007). Targeted therapy for renal cell carcinoma: A new treatment paradigm. Proc. (Bayl. Univ. Med. Cent.).

[155] Eggener S.E., Yossepowitch O., Pettus J.A., Snyder M.E., Motzer R.J., Russo P. (2006). Renal cell carcinoma recurrence after nephrectomy for localized disease: Predicting survival from time of recurrence. J. Clin. Oncol..

[156] Eggener S.E., Yossepowitch O., Kundu S., Motzer R.J., Russo P. (2008). Risk score and metastasectomy independently impact prognosis of patients with recurrent renal cell carcinoma. J. Urol..

